# The legacy of raw milk storage temperature is associated with cheese microbiome composition, notwithstanding pasteurization and starter addition

**DOI:** 10.1093/femsle/fnag046

**Published:** 2026-04-16

**Authors:** Lucia Giagnoni, Saptarathi Deb, Alessandra Tondello, Michele De Noni, Matteo Borella, Piergiorgio Stevanato, Alessio Cecchinato, Andrea Squartini, Carlo Spanu

**Affiliations:** Department of Veterinary Medicine, University of Sassari, Via Vienna, 2, 07100 Sassari, SS, Italy; Department of Agronomy, Food, Natural Resources, Animals and Environment, DAFNAE, University of Padova, Viale dell’Università 16, 35020 Legnaro, PD, Italy; Department of Agronomy, Food, Natural Resources, Animals and Environment, DAFNAE, University of Padova, Viale dell’Università 16, 35020 Legnaro, PD, Italy; Department of Agronomy, Food, Natural Resources, Animals and Environment, DAFNAE, University of Padova, Viale dell’Università 16, 35020 Legnaro, PD, Italy; Latteria Montello S.p.A., Via Fante d’Italia 26, 31040 Giavera del Montello, TV, Italy; Department of Agronomy, Food, Natural Resources, Animals and Environment, DAFNAE, University of Padova, Viale dell’Università 16, 35020 Legnaro, PD, Italy; Department of Agronomy, Food, Natural Resources, Animals and Environment, DAFNAE, University of Padova, Viale dell’Università 16, 35020 Legnaro, PD, Italy; Department of Agronomy, Food, Natural Resources, Animals and Environment, DAFNAE, University of Padova, Viale dell’Università 16, 35020 Legnaro, PD, Italy; Department of Agronomy, Food, Natural Resources, Animals and Environment, DAFNAE, University of Padova, Viale dell’Università 16, 35020 Legnaro, PD, Italy; Department of Veterinary Medicine, University of Sassari, Via Vienna, 2, 07100 Sassari, SS, Italy

**Keywords:** fresh cheese, milk refrigeration temperature, thermal abuse, 16S metabarcoding, psychrotrophs, pseudomonads

## Abstract

Using DNA metabarcoding, we assessed the relative impact of two variables: (a) raw milk storage temperature and (b) cheese maturation duration, on a semi-fresh cheese bacteriome composition under authentic factory-scale industrial conditions. The study is compared to a prior literature report run at a high-quality milk facility, whilst in the present case, milk from an average-quality farm, better reflecting typical local standards, was used. We tested three milk storage temperatures (4°C, 7°C, 9°C) and sampled cheese at six maturation stages (0, 10, 25, 30, 45, and 60 days). Results showed that raw milk storage temperature was the variable most strongly associated with microbial community composition across the sampled stages, exceeding the variation associated with maturation time. Even the pasteurization step and the addition of a *Streptococcus thermophilus* starter culture did not erase the microbial ‘memory’ of initial milk conditions. Interestingly, the cheese bacterial community shaping associated with different milk tank temperatures was moreover compositionally uncoupled from the dominant taxonomical pattern of the starting milk. Additionally, the study provided insights into balancing milk quality and storage temperatures to prevent spoilage by psychrotrophic pseudomonads. Under the conditions tested here, the findings suggest that the 4°C storage benchmark may warrant re-evaluation.

## Teaser

Bacterial community composition in cheese can be very different from that of the starting milk, but it is strongly affected by the temperature at which milk is stored before cheesemaking. This entanglement occurs in spite of the abatement of milk microbiota by pasteurization and of the massive addition of a live microbial starter inoculum.

## Introduction

In dairy value chains, quality and characteristics of cheese depend on many factors. Variations in raw milk storage temperature and cheese maturation duration are important variables associated with cheese quality and microbiome composition. We demonstrated this in prior experimental tests conducted at real scale within a major cheesemaking factory (Giagnoni et al. 2023). Raw milk transformation into matured cheese involves complex interplays of biological, chemical, and environmental processes. Temperature is a critical environmental variable significantly influencing these processes, impacting cheese shelf life and sensory properties of the final product, as well as its microbiota (Falardeau et al. 2019, Zago et al. 2022). Controlling the milk tank temperature is essential for preserving milk quality before cheese production, yet this variable is understudied because the temperature of +4°C is commonly assumed as the best compromise and has become an established standard. Fluctuations in milk tank temperature, referred to as unintended thermal abuse, can initiate biochemical reactions that alter milk composition, potentially jeopardizing subsequent cheese manufacturing steps. Additionally, methods of affinage, the process of imparting specific flavors and textures to cheese, profoundly shape its final taste profile (Mayo et al. 2021). Therefore, understanding the intricate relationship between milk tank temperature variations, cheese maturation time, and resulting cheese quality is crucial for dairy industry professionals, researchers, and consumers. This knowledge empowers cheese producers to optimize their processes, enhance cheese shelf-life, and enrich the sensory experience for consumers, thereby contributing to the overall appreciation of this beloved dairy product.

In dairy microbiology, the temperature at which milk is refrigerated before pasteurization impacts overall microbial diversity, governing the delicate balance between bacterial taxa. Psychrotrophic bacteria, thriving at low temperatures, are important players among microbial groups in this context. The relative proportion of psychrotrophs on the total milk bacterial population is critical, as it dictates quality and characteristics of the final dairy products. Some microorganisms produce thermostable extracellular enzymes, as proteases or lipases, which can negatively impact cheese yield, stability, nutritional value, and other processing-related factors (Cousin and Marth 1977, Cousin 1982, Ercolini et al. 2009, Decimo et al. 2014, Lorenzo et al. 2018). Psychrotrophs are sensitive to pasteurization, except for a few gram-positive species that are also thermoduric. Nonetheless, protease and lipase enzymes can remain active after heat treatments used to kill vegetative forms (Oliveira et al. 2015).

Traditionally, 4°C has been the gold standard for milk refrigeration due to its broad inhibitory effect on bacterial growth. While this temperature is effective in controlling the proliferation of mesophilic organisms, it presents an undiscussed conundrum. In this chilled environment, certain cold-tolerant genera exhibit proteolytic activity. When these bacteria proliferate, they can adversely affect processes, as curd clotting and cheese maturation (Lorenzo et al. 2018).

The delicate balance of inhibiting undesirable growth while preserving the integrity of beneficial microbial communities raises intriguing questions. Understanding the dynamics connecting milk refrigeration temperature, psychrotrophic taxa proliferation, and their subsequent impact on cheese production is important, and direct collaboration with the industry is critical to obtain feasible applications.

The present study, in comparison to a different report (Giagnoni et al. 2023), was commissioned by a major cheese producer and conducted as full-scale in-factory trial. The cited article had tested a broad temperature range on a model farm. The present study is instead focusing on exposing raw milk to temperatures that are commonly considered abusive (above 4°C) during refrigeration to evaluate effects on microbial composition of semi-fresh cheese during maturation. The distinctive elements of the present experimentation compared to the above reference were: (1) testing milk from an average milk hygiene quality dairy farm instead of a high-quality farm, to more accurately represent the regional reality of standard cheesemaking plants, (2) focusing on a finer range of temperatures within the previously explored span by concentrating on closer thermal ramping steps (4°C, 7°C, 9°C), and (3) analyzing cheese microbiome composition through a prolonged maturation period by extending it up to 60 days. Experiments involved three milk storage temperatures prior to pasteurization and cheese production. 16S-based metabarcoding allowed exploring taxonomic identities and relative abundance of bacteria at six cheese maturation stages. The study focused on identifying taxa relevant to the dairy process, including thermophilic, thermoduric, and psychrotrophic microorganisms that could compromise milk quality and safety.

The aim of this investigation was to assess how two unconventional milk refrigeration temperatures prior to pasteurization were associated with the microbiome composition of a semi-fresh cheese, and to evaluate whether the usual benchmark of 4°C remained the most suitable compromise for overall dairies, or reconsiderations could apply depending on their supply chain hygienic class level.

The experiment was conducted at the plant of a collaborating dairy company and followed a systematic procedure involving incremental thermal changes reaching three different temperatures. To investigate long-term effects of milk exposure to these increments on the resulting product, the temporal dynamics of cheese bacterial communities were evaluated throughout six maturation times, from time zero up to two months. The working hypothesis was that the standard reference temperature of 4°C might not necessarily be the most suitable choice for producing semi-fresh cheeses for all dairy chain facilities.

## Materials and methods

### Experimental setup and sample preparation

The Latteria Montello dairy company, based in Giavera del Montello, northeastern Italy, arranged a specific thermal ramping schedule for the milk storage tank at one of their partner farms. They subsequently performed the cheese-making and maturation storage operations in their industrial facilities. Milk and cheese samples were then delivered to our laboratories for DNA-based analyses. The standard bulk milk cooling tank (Mod. SE 4000, Danfoss Italia, Venice, Italy) with a capacity of 4000 l was used to store raw cow’s milk. The standard operating temperature of the bulk tank was 4°C. Two temperature increases were applied to reach 7°C and 9°C to simulate thermal abuse and evaluate its effect on the microbiological community of a semi-fresh cheese during ripening. Milk was stored at each temperature for 24 h. To allow time for temperature adjustment and for the microbiota to reach equilibrium rather than experience a sudden change in the bulk tank’s environment, each temperature was maintained for 12 days before the experimental incubation and subsequent sampling. During this time, milk was collected and replaced daily with fresh milk as part of the usual routine. After such a temperature adaptation period, samples of the milk in the tank were collected. Clarifying this step, although milk was removed from the tank daily and the standard cleaning and sanitizing routine was applied, the cheese-making process for our three series of experiments only used the milk collected on the twelfth day after storage tank conditioning began at each temperature (whereby the loads collected on each of the preceding 11 days were discarded).

The milk had an average water content of 87.2%, 5.1% carbohydrates, 3.65% lipids, and 3.28% proteins. Regarding the routine microbiological cultural analyses, total bacterial counts were obtained by plating milk dilutions on sugar-free FIL-IDF Colony Count Agar (Merck Inc., Rahway, NJ, USA). Coliforms were enumerated by culturing on chromogenic coliform agar (Biolife Corp., Milan). The microbiological profile of milk stored at three different temperatures, in terms of total bacteria (CFU/ml), was as follows: 95 000 (4°C), 150 000 (7°C), and 1 500 000 (9°C). Regarding the coliforms, the CFU/ml values were 8000 (4°C), 25 000 (7°C), and 70 000 (9°C).

The dairy facility received the milk and pasteurized it at 75°C for 30 s. Aliquots of 50 ml of milk were taken before and after pasteurization. These were promptly frozen at −20°C and used as a starting milk control for metabarcoding analyses. “Caciotta-style” semi-fresh cheese from the same milk was prepared in the dairy plant. The choice made by the company was to produce a very plain cheese of basic type that does not correspond to those that are in their presently branded and marketed line. This was deemed preferable since, having to assess novel protocol variations, such as the milk storage temperature, using a very neutral standard prototypic cheese, allowed to inspect its behaviour during the coagulation and cheesemaking, with less process-related factors. The protocol was the following: after chilling the pasteurized milk to 40°C, it was quickly poured into a vat containing rennet to coagulate the casein and a commercial acidifying starter culture (*Streptococcus thermophilus*). The rennet used was microbial, derived from the fungus *Rhizomucor miehei*, at a dosage of 1500 International Milk-Clotting Units (IMCU) per 100 l of milk. The starter culture added was 200 g, with a titer of 1×10¹¹ cells per gram. Coagulation occurred within 10 to 15 min, and acidification took 2 h. No physical differences were observed in relation to the initial milk tank storage temperature for these steps. Mild stirring was part of the whey syneresis process. Once the curd had formed, it was cut into cubic pieces with 3-cm sides using a curd cutter. The whey was separated from the curd through the perforated colander. It was drained and placed into rectangular molds until the pH reached 5.5 (∼2 h). The molds were turned over twice during this time to promote mass homogeneity. The molds were kept for 30 min at 6°C–7°C in a 20% NaCl brine solution before being transferred to the ripening chamber at 4°C. For each of the different temperature experiments, the content was extracted, consisting of a 12-kg cheese block with the following characteristics (w/w): 48%–52% water, 24%–26% fat, 22%–24% protein, 0.1%–0.5% lactose, 0.5%–0.7% NaCl, and 0.98–0.99 water activity (a_w_). For this purpose, two 1.5 kg portions of cheese were taken from each block. From each portion, six 250-gram portions were prepared: one to be analyzed immediately (time 0), and the other five to be stored for maturation at 4°C and removed after 10, 25, 30, 45, or 60 days. At each sampling time, the portions were stored at −20°C and subsequently processed in parallel for DNA-based analysis.

A total of 36 cheese samples were analyzed, coming from three temperature regimes (4°C, 7°C, and 9°C), with two replicates tested at each of the six sampling times (0, 10, 25, 30, 45, and 60 days).

For a better visualization of the experimental workflow, see also the graphical abstract.

### DNA extraction

One gram of thawed cheese, cut with a sterile scalpel and weighed on a laboratory scale using a sterile petri dish as a tray, was transferred into a 2-ml Eppendorf tube for each sample. Tubes were centrifuged at 21 000 g for 10 min. The supernatant was discarded, and 350 μl of buffer (guanidine thiocyanate 0.12 M, Qiagen, Hilden, Germany), along with a 5 mm tungsten bead, were added to the pellets. Homogenization took place in a TissueLyser II (Qiagen, Hilden, Germany) at 30 Hz for 5 min, repeated twice with a 1-min break. Samples were centrifuged at 21 000 g for 10 min to remove the floating fat layer, which was pipetted out from the top. The residual aqueous phase underwent enzymatic treatment with 20 μl of a 20 mg/ml lysozyme solution (Thermo Fisher Scientific, Waltham, MA, USA). The solution was mixed by vortexing 3 s and incubated at 37°C for 30 min. Twenty microliters of a 20 mg/ml proteinase K solution (Invitrogen, Carlsbad, CA, USA) was added. Samples, which were incubated at 50°C for 30 min. The final steps involved vortexing the samples for 3 s, centrifuging them at 2500 g for 6 min, collecting the supernatant, and purifying DNA on a BioSprint 96 automated workstation (Qiagen, Hilden, Germany). Twenty microliters of MagAttract magnetic beads suspension (Qiagen, Hilden, Germany) and 200 μl of isopropanol were added to an S-block containing sample supernatants and placed into the instrument. The protocol involved sequentially: one S-block containing 500 μl of RPW buffer (guanidine hydrochloride at 1.31 M), two plates containing 500 μl of 96% ethanol each, an additional S-block containing 500 μl of a 0.02% Tween-20 solution, and a flat-bottom 96-well plate containing 80 μl of nuclease-free water eluting the DNA. Final DNA concentrations were determined using a Qubit 3.0 Fluorometer and the Qubit™ DNA HS Assay Kit from Thermo Fisher Scientific (Waltham, MA, USA).

### Metabarcoding of the bacterial 16S rRNA gene

The 16S Ion Metagenomics Kit (Thermo Fisher Scientific, Waltham, MA, USA) was used for library preparation. It included an initial PCR amplification using two separate sets of primers to amplify hypervariable regions (V2, V4, V8, and V3, V6-7, V9). Library preparation and PCR followed the program described by Giagnoni et al. (2023). Amplicons were measured and combined to achieve a final concentration of 30 ng/l. The Ion Express Barcode Kit and the Ion Xpress Plus 9 Fragment Library Kit (Thermo Fisher Scientific, Waltham, MA, USA) were used for barcode ligation in the process. Library quantification used a Qubit™ DNA HS Assay Kit on a Qubit 3.0 Fluorometer. Libraries were pooled at a final concentration of 100 pM. Following the manufacturer’s instructions, the samples were processed using an Ion 520™ and an Ion 530™ Kit—OT2 400 bp (Thermo Fisher Scientific, Waltham, MA, USA). The samples were placed onto an Ion 520 chip, and the sequencing run was performed using an Ion™ GeneStudio™ S5 System, both from the same manufacturer.

### Bioinformatics and statistics

The cutadapt v3.4 software (Martin, 2011)) and the QIIME2 v2021.4 software (Bolyen et al. 2019) were used to process raw reads and eliminate primers by trimming 20 base pairs from both ends of the reads. The qiime dada2 plugin was used to denoise and dereplicate high-quality reads into amplicon sequence variants (ASVs). The “qiime alpha-diversity” plugin was used to create an alpha-rarefaction map to evaluate sequencing depth. The SILVA SSU v138.1 database (Quast et al. 2012)) was used for taxonomic attribution of ASVs. We normalized the abundance values using log transformation to account for sequencing depth. The data matrix was analyzed with Past 4.11 software (Hammer et al. 2001)) to calculate ecological diversity indexes, including Chao1, the Shannon-Wiener H value, Simpson’s 1-D, and evenness. To minimize sequencing errors, the metagenomic data were processed using log base-e transformation and filtered to remove reads with an abundance of less than two. The relative abundance percentage of the major taxa at genus level, Shannon diversity ecological index, and the nonparametric Kruskal–Wallis statistical test with Dunn’s Post Hoc and FDR correction were used to determine the significance of alpha diversity differences using the Microbiome Analyst online tool (https://www.microbiomeanalyst.ca/). Non-metric multidimensional scaling (NMDS), based on Bray–Curtis dissimilarities, was used to calculate beta diversity among samples. Pairwise comparisons between groups were performed using the Wilcoxon signed-rank test with a significant *P*-value of less than 0.05, using R software version 4.3.1 (https://www.r-project.org/). Differential representation analysis of the major taxa at genus level in the cheese sample communities was inspected using the SHAMAN online utility (https://shaman.pasteur.fr/). We performed functional prediction inference from metabarcoding taxonomy using FAPROTAX software (Louca et al. 2016) to predict the main metabolic processes of the microbial communities. Sequences have been deposited in GenBank under BioProject code PRJNA1395772.

## Results

A total of 6 180 197 paired-end raw reads were generated by sequencing the bacterial 16S rRNA gene. On average, 159 385 reads were obtained per sample, ranging from 33 290 to 275 821. Regarding the initial microbiological state of milk stored at standard temperature (4°C) or the two investigated elevated temperatures (7°C and 9°C), the DNA sequencing data are presented in Table 1, in detail, and as stacked bar histograms in Fig. S1 of the Supplementary Material.

**Table 1:** Sequencing results in milk samples prior to pasteurization.

Milk 4°C	reads	%	Milk 7°C	reads	%	Milk 9°C	reads	%
*s__Pseudomonas_fragi*	21 947	44.394	*g__Achromobacter*	20 374	34.447	*s__Lactococcus_lactis*	22 804	40.558
*g__Pseudomonas*	12 514	25.313	*s__Pseudomonas_fragi*	20 356	34.417	f__Enterobacteriaceae	11 300	20.097
*g__Achromobacter*	5594	11.315	*g__Pseudomonas*	5763	9.744	*g__Lactococcus*	6418	11.415
*s__Pseudomonas_sp*.	5301	10.723	f__Alcaligenaceae	5330	9.012	*g__Enterobacter*	2101	3.737
f__Alcaligenaceae	1641	3.319	*s__Pseudomonas_sp*.	4599	7.776	*g__Achromobacter*	1889	3.360
*g__Acinetobacter*	698	1.412	*g__Acinetobacter*	868	1.468	*s__Streptococcus_parauberis*	1662	2.956
*s__Lactococcus_lactis*	451	0.912	*s__uncultured_bacterium*	646	1.092	*g__Pseudomonas*	1599	2.844
*s__Acinetobacter_lwoffii*	303	0.613	*s__Streptococcus_salivarius*	275	0.465	o__Enterobacterales	968	1.722
*s__uncultured_bacterium*	161	0.326	*s__Acinetobacter_lwoffii*	120	0.203	*s__Serratia_marcescens*	946	1.682
*g__Alkanindiges*	160	0.324	*s__Lactococcus_lactis*	113	0.191	*g__Acinetobacter*	898	1.597
f__Moraxellaceae	126	0.255	*g__Microbacterium*	100	0.169	*g__Citrobacter*	833	1.482
s__uncultured bacterium	126	0.255	*s__Acinetobacter_johnsonii*	63	0.107	*s__Citrobacter_sp*.	734	1.305
*s__Streptococcus_salivarius*	96	0.194	*s__Burkholderia_sp*.	37	0.063	*g__Serratia*	603	1.072
*g__Lactococcus*	72	0.146	f__Moraxellaceae	35	0.059	f__Alcaligenaceae	546	0.971
*s__Burkholderia_sp*.	43	0.087	*g__Halomonas*	28	0.047	*s__Pseudomonas_fragi*	457	0.813
*g__Microbacterium*	24	0.049	*g__Sphingomonas*	25	0.042	*g__Streptococcus*	433	0.770
*g__Enhydrobacter*	18	0.036	*g__Cutibacterium*	22	0.037	*s__uncultured_bacterium*	403	0.717
*g__Burkholderia*-C-P *	12	0.024	*s__Streptococcus_uberis*	20	0.034	*s__Pseudomonas_sp*.	258	0.459
o__Enterobacterales	11	0.022	*g__Alkanindiges*	19	0.032	*s__Acinetobacter_lwoffii*	215	0.382
*g__Corynebacterium*	10	0.020	s_uncultured _bacterium	19	0.032	*s__Hafnia_alvei*	185	0.329
*s__Staphylococcus_epidermidis*	10	0.020	*s__Lactobacillus_delbrueckii*	17	0.029	*g__Rahnella1*	172	0.306
*g__Streptococcus*	8	0.016	*g__Rheinheimera*	16	0.027	*s__Streptococcus_uberis*	137	0.244
*g__Enterobacter*	7	0.014	*g__Corynebacterium*	15	0.025	*g__Klebsiella*	118	0.210
*g__Serratia*	6	0.012	*s__Lactobacillus_helveticus*	12	0.020	*g__Alkanindiges*	110	0.196
*g__Cutibacterium*	5	0.010	*g__Enterococcus*	11	0.019	f__Moraxellaceae	72	0.128
Total reads	98 874		Total reads	118 172		Total reads	112 452	
N. of taxonomic names	84		N. of taxonomic names	128		N. of taxonomic names	90	
N. of different ASV	245		N. of different ASV	383		N. of different ASV	337	

The top 25 taxonomical annotations assigned from the bioinformatics pipeline are shown. Number of sequence reads and percentage over the sample total are reported. Reads data are the mean of two replicates. Values are rounded up to integers. The total number of amplicon sequence variants (ASV) and that of the corresponding different taxonomical names are indicated. *Burkholderia-C-P: *Burkholderia-Caballeronia-Paraburkholderia* group. The letters preceding taxa names indicate the rank (o: order, f: family, g: genus, s: species). ^1^Note: the sequencing of milk after pasteurization yielded consistently similar results (data not shown), which is expected by the fact that the metabarcoding technique is based on DNA thermal amplification, and thus pasteurization only impairs cells vitality but does not affect their DNA.

At the standard refrigeration temperature of 4°C, the milk was dominated by the psychrotrophic genus *Pseudomonas*, with three instances, which together accounted for over 80% of the microbiome sequence abundance. There were also lower instances of *Achromobacter*, slightly above 11.3%, as well as several minor entries. Increasing the temperature by three degrees profoundly changed the patterns of the same tank due to the rise of *Achromobacter*, which surpassed *Pseudomonas* and became the dominant taxon with over 34% presence. The increased temperature was also accompanied by a significant increase in total diversity, with the number of taxa annotations rising from 84 to 128, and the number of sequence variants increasing from 245 to 383. When set and conditioned at a two-degrees-higher temperature (9°C), the reservoir underwent another major successional shift, with a dominance of acidifying lactic acid bacteria (*Lactococcus lactis*) up to 40.5%, as well as an abundance of enteric bacteria, which are associated to warm-blooded animals. The diversity remained high with no evident changes compared to that attained at 7°C. The striking differences in raw milk composition made the planned follow-up particularly intriguing, as it allowed assessment of the extent to which these differences were reflected in the microbial communities of cheese produced from each type of milk. Of particular interest was verifying whether a homogenizing selection (i.e. a process promoting a similar assembly outcome) would prevail in the resulting cheese communities due to milk pasteurization at 75°C and the addition of the *Streptococcus thermophilus* starter culture. Another possible outcome was that, despite these strong constraints, the cheese microbial communities would maintain specificities inherited from the initial storage temperature to which the milk had been exposed for one day throughout the 60-day cheese maturation process.

**Figure 1:**
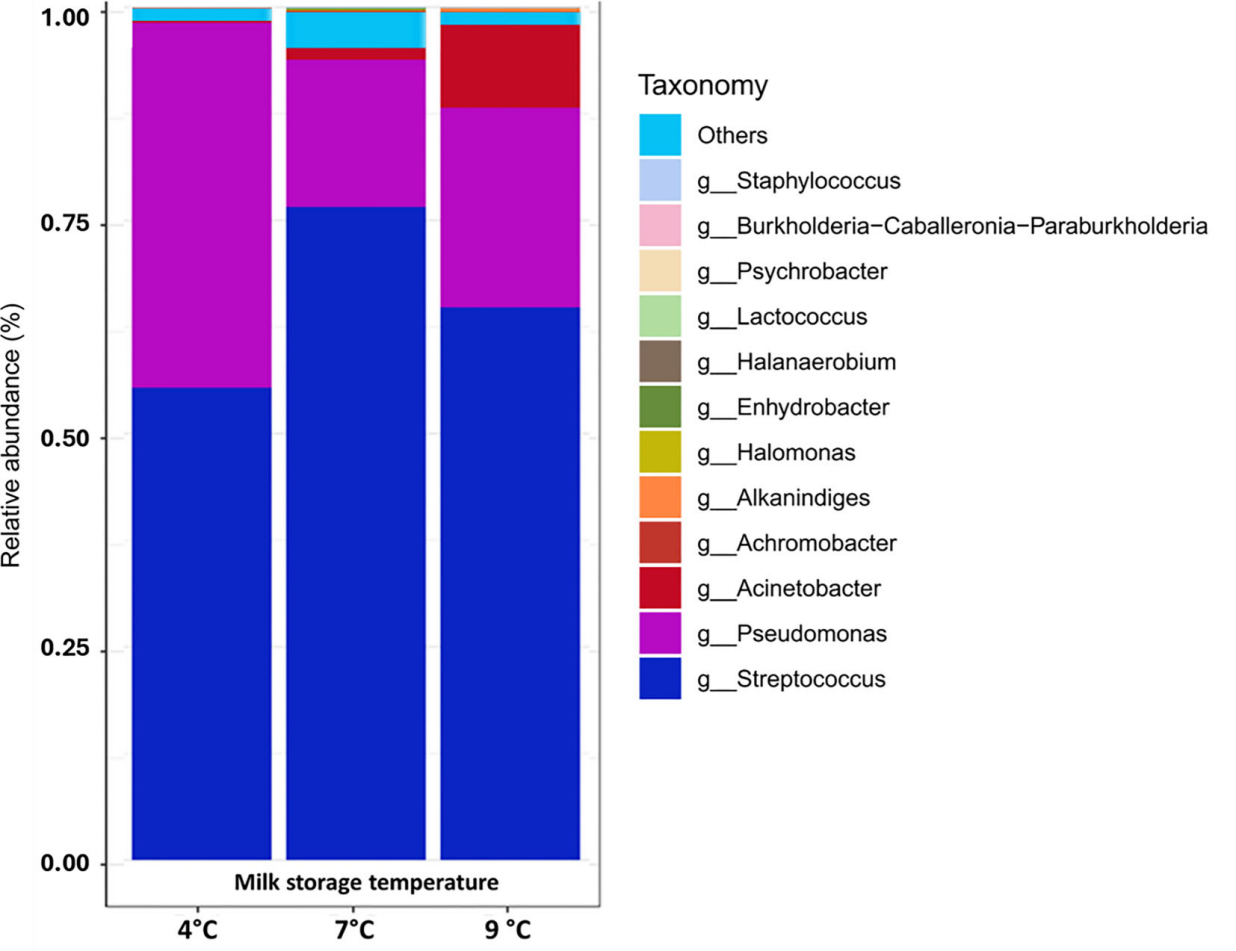
Relative abundance of the twelve most abundant bacterial genera in cheese as a function of milk storage temperature (4°C, 7°C, 9°C). The stacked bars report the mean values across all timepoints. For the significance of differences between taxa in relation to each of the three temperature contrasts, see Fig. 6.

Concerning the relative abundance of bacteria in cheeses produced from raw milk stored at three different temperatures (4°C, 7°C, and 9°C) before pasteurization, the majority were Firmicutes (63.51% of sequences), followed by Proteobacteria (36.43%) and Halanaerobiaeota (0.03%) at much lower abundance. The Firmicutes predominantly belonged to the Bacilli class, with the Lactobacillales order and the *Streptococcus* and *Lactococcus* genera dominating the cheese biota. The observed Proteobacteria were mainly from the Gammaproteobacteria class and included the genera *Pseudomonas, Acinetobacter*, and *Aeromonas*, along with various members of the Enterobacteriales order. Figure 1 shows the dominant genera proportions. As expected, *Streptococcus* was prevalent because it belongs to the starter culture used for cheesemaking. This culture was added to each type of milk equally after pasteurization and is therefore independent of the prior milk thermal storage variable.

Nevertheless, in metabarcoding, the relative abundance of any species is determined both by its own behavior and by the dynamics of other taxa. The primary competitor of the starter appeared to be the *Pseudomonas* genus. Although *Streptococcus* accounted for nearly 80% of the sequence reads in cheese from milk stored at 7°C, this value was lower than 60% when the standard temperature (4°C) was used. Essentially, the psychrotrophic and potentially spoiling *Pseudomonas* appeared to be more competitive than the starter strain when milk stored at the lowest temperature was processed. But the *Streptococcus* starter, although the colder temperature was unfavorable for it, also suffered the progressive, temperature-driven rise of other taxa that culminated at the warmest one. This was mostly due to *Acinetobacter*, which remained relatively constant throughout the three types of milk (Table 1) but reached its maximum percentage in cheese obtained from milk stored at the trial’s warmest endpoint temperature (9°C).

**Figure 2:**
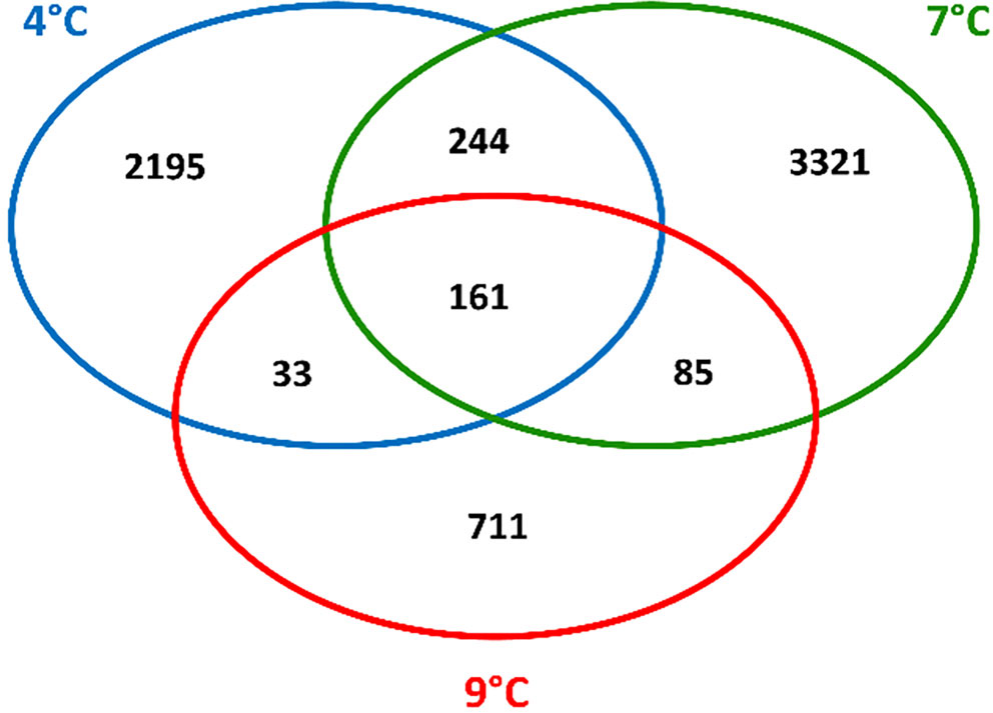
Venn diagram representing the numbers of amplicon sequence variants (ASVs) detected in cheese and belonging either to the core microbiome or to shared or unique sets. Data report the variants found in cheese, collected at any maturation stage and made from milk originally stored at each of three temperature values of 4°C, 7°C, and 9°C.

From a quantitative perspective, the cheese microbiota showed a limited set of common core taxa and a large number of specific ones linked to the milk storage temperature. Figure 2 shows the overlapping patterns.

**Figure 3:**
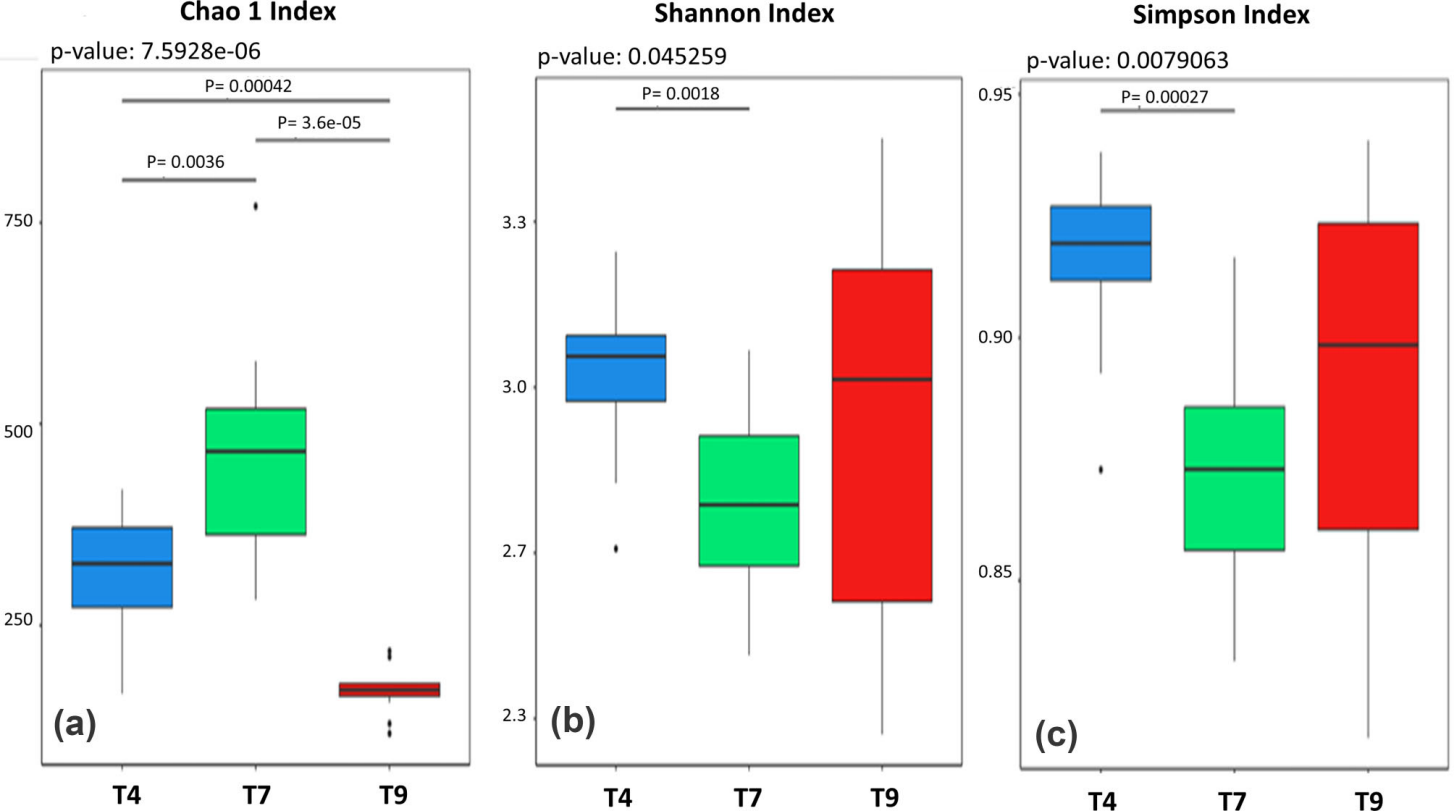
Alpha-diversity analysis in cheese samples by the Chao1 (3a) Shannon (3b), and Simpson (3c) ecological indices as a function of milk storage temperature (T) at 4°C, 7°C, 9°C prior to pasteurization. On top of each image, the Kruskal–Wallis test p-value for the group is reported, while inside the frame, the significant *P*-values between the encompassed pairs of sample groups stemming from the Wilcoxon signed-rank test are indicated.

Cheese made from milk stored at 4°C had 2633 variants, while cheese made from milk stored at 7°C had 3811 variants. Only 990 variants occurred in cheese made from milk stored at 9°C. Only 161 ASVs were common to products made from milk of any of the three temperatures. Therefore, the core microbiome analysis revealed that cheese from milk stored at 7°C prior to pasteurization had the highest richness of amplified sequence variants. This finding is also evident in the alpha-diversity plot of the Chao1 index at the species level. Significant differences between communities at each temperature are shown in Fig. 3a.

In contrast, both species diversity indices, Shannon–Wiener H (Shannon 1948) and Simpson’s 1-D (Simpson 1949) showed significance only for the differences between the milk storage temperatures of 4°C and 7°C (Fig. 3b and c), with higher values for the former. Additionally, values were very dispersed (wider error bars) for the two higher temperatures, unlike the Chao 1 index. These differences align with the nature of the two indices, which essentially express a ratio of richness to uniformity of distribution, with more emphasis on richness for the Shannon index and more on uniformity for the Simpson index.

**Figure 4:**
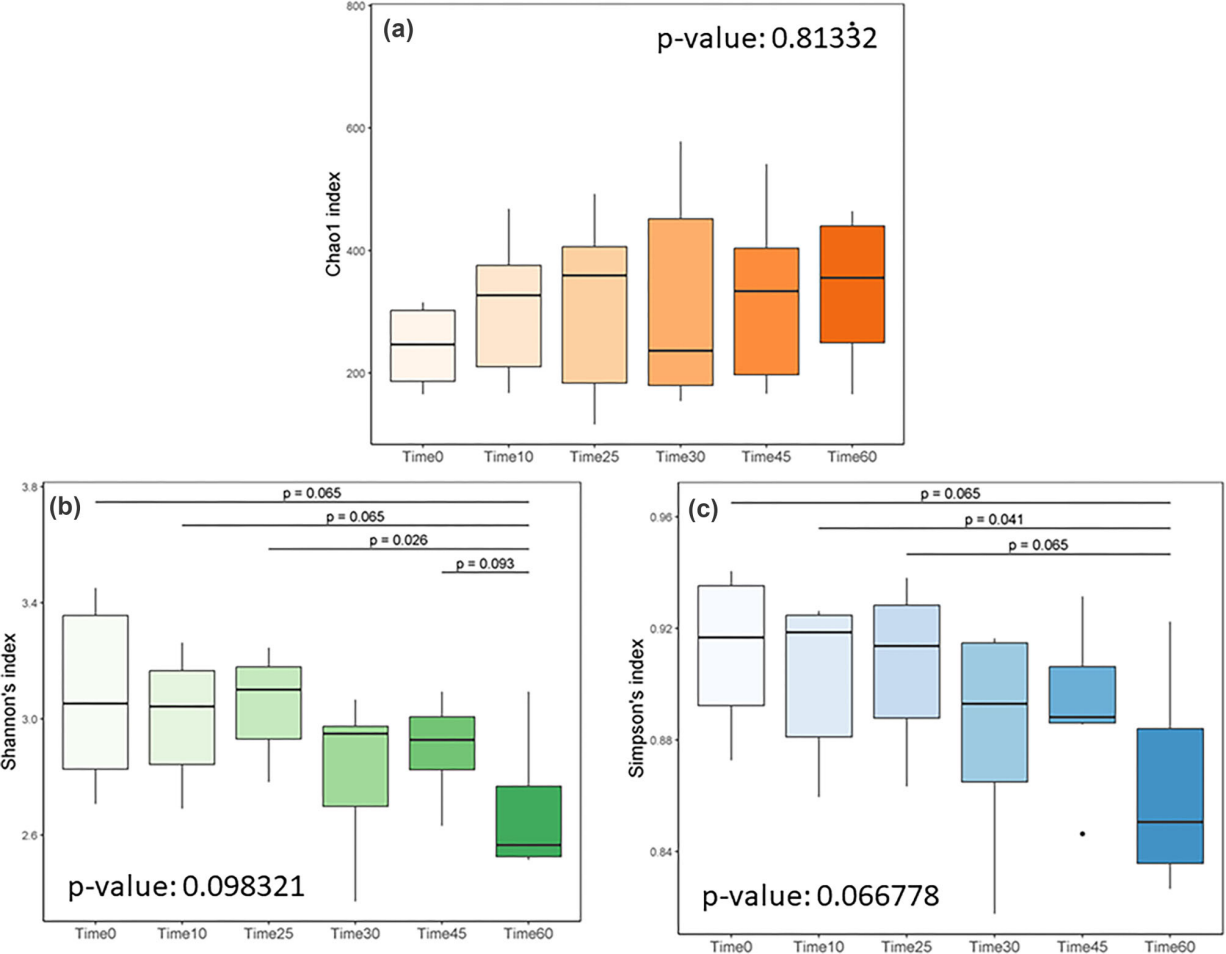
Alpha-diversity analysis with (a) Chao1, (b) Shannon, and (c) Simpson indices as a function of cheese communities sampled through the six maturity stages. Inside each image, the Kruskal–Wallis test *P*-value for the group is reported, while over the different bars across the boxplots, the p-values (only those for *P* < 0.1), between the encompassed pairs of sample groups stemming from the Wilcoxon signed-rank test are indicated.

Regarding alpha diversity as a function of cheese maturity through six stages, the Chao 1 index showed no significant differences, but a slight increasing trend (Fig. 4a). The Shannon and Simpson indexes, however, revealed a gradual decrease in levels during maturation. In specific paired comparisons, this decrease was significant at *P* < 0.05 using the Wilcoxon signed-rank test (Time 25 vs. Time 60 for the Shannon index and Time 10 vs. Time 60 for the Simpson index). In any event, the significance levels of differences between cheese communities at different maturation times were moderate compared to those related to milk temperature, as discussed above regarding Fig. 2.

To appreciate the strong effects of milk temperature on the bacterial community of cheese compared to the moderate effects due to maturation stages, these effects have been compiled in three panels in Supplementary Material, Figure S2.

**Figure 5:**
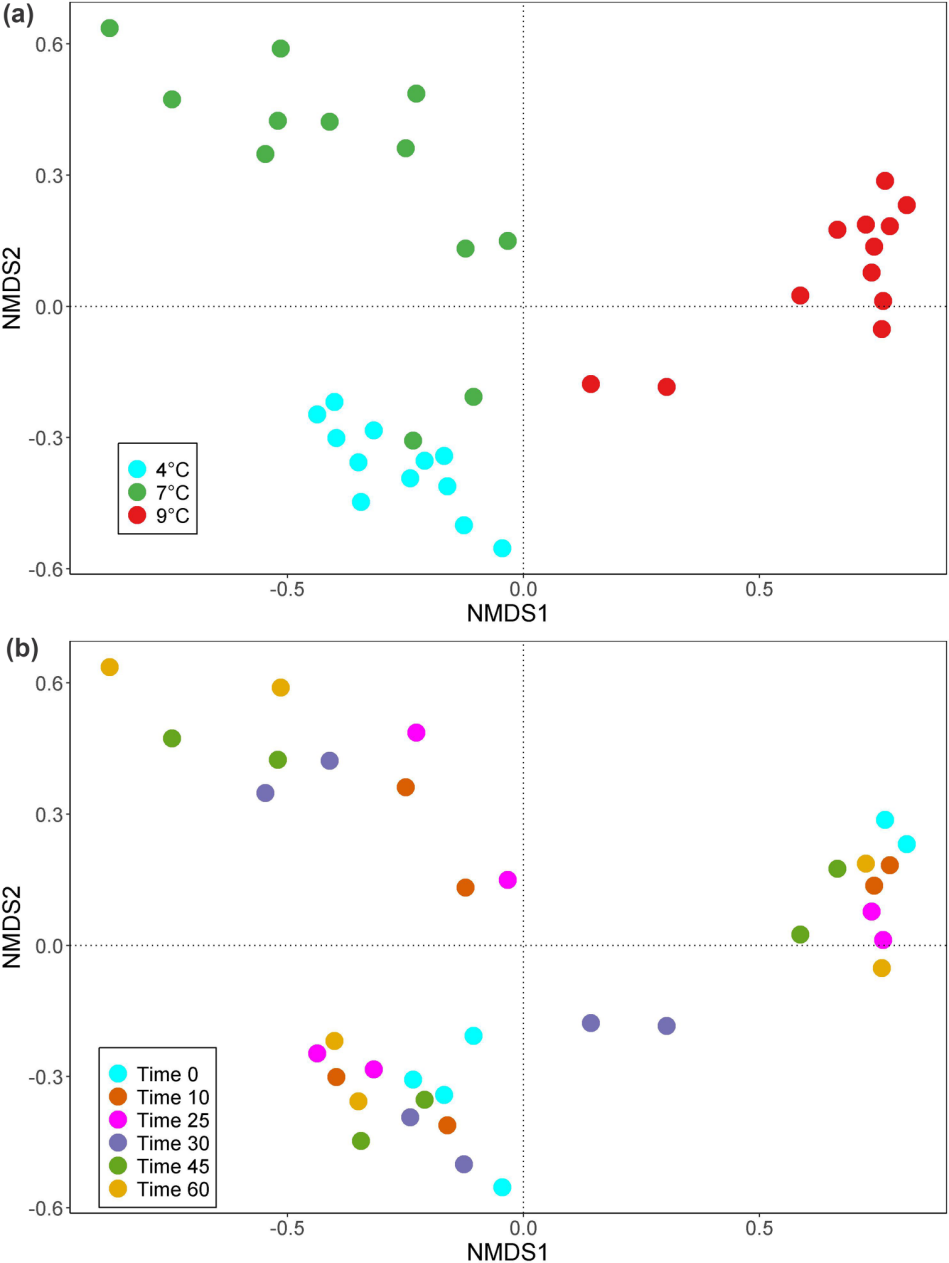
Non-metric multidimensional scaling analysis (NMDS) of the cheese bacteria community data as a function of (a) raw milk storage temperature at 4°C, 7°C, 9°C before pasteurization, and (b) cheese maturation stage.

The multivariate non-metric multidimensional scaling plots in Fig. 5 provide the clearest visualization of the strong association between initial milk temperature and subsequent cheese bacterial community structure within this dataset. The NMDS drawn from Bray–Curtis community distances yielded the ordination displayed in this figure. The low stress factor value (0.083499) indicates a good representation of sample clustering in the reduced dimensions of this scaling analysis. Sample labels of the bacterial communities retrieved from each cheese block are indicated by initial milk temperature (panel A) and maturation time (panel B). The evident clustering into three well-resolved clouds was strongly associated with the temperature of the milk before pasteurization and before starter addition. This pattern is consistent with a relationship between pre-cheesemaking milk conditions and subsequent cheese microbiome differences. The known variable in this flow was the storage tank refrigeration temperature, although additional environmental contributions, such as e.g. milk load of the day, cannot be excluded.

Conversely, no evidence of time-related clustering was observed regarding the cheese maturation period; its effect on community separation was minimal compared to that from milk temperature.

Using a PERMANOVA to verify pattern robustness, we found significant separation among milk temperature groups (F-value: 20.418, R-squared: 0.55306, *P*-value: 0.001), but not among different cheese maturation times (F-value: 1.11, R-squared: 0.15612, *P*-value: 0.356).

The identities of the taxonomic groups on which such ordination relies are presented in full complexity in Supplementary Material Fig. S3 heat trees.

**Figure 6:**
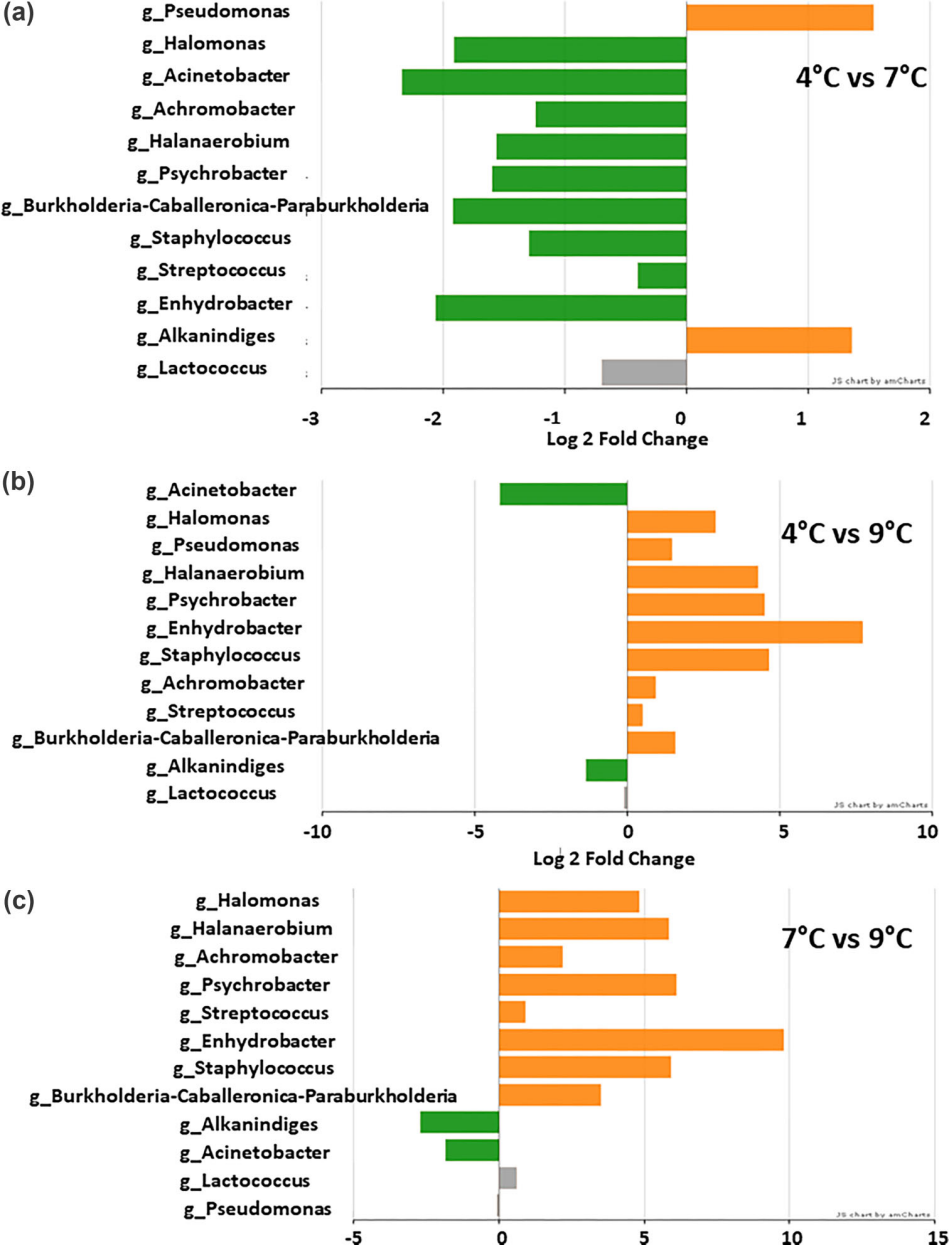
Genera displaying significantly different representations (*P* < 0.05) in the cheese samples communities upon comparisons between pairs of temperatures. a: 4°C vs 7°C, b: 4°C vs. 9°C; c: 7°C vs 9°C. The log 2 fold change extent is plotted on the horizontal scale. Over-represented taxa (in the first temperature of each contrast) are indicated by rightward orange bars, while under-represented ones by leftward green bars, in both cases significantly for *P* < 0.05. Non-significant changes are shown with grey bars.

The list of genera displaying differential representation in the three temperature contrasts is shown in Fig. 6.

Inspecting the identities of the taxa involved in these reciprocal fluctuations, which are for the most part statistically supported, the differentially-represented genera emerging from the three pairwise temperature contrasts are visualized. From 4°C to 7°C, *Pseudomonas* falls while several other Gammaproteobacteria, Betaproteobacteria, and Gram-positives rise, opportunistically exploiting the increased temperature. However, their retreat is equally evident when passing from 7°C to 9°C, at which *Alkanindiges* and *Acinetobacter* increase.

Finally, running the function-predictive tool FAPROTAX on taxonomy data of the cheese communities inferred a series of categories, as depicted in the heatmap shown in Supplementary Material Fig. S4. FAPROTAX uses database knowledge associated with taxonomic assignments to list functions reported to be featured by the identified taxa. Besides confirming the temperature-driven clustering of the dendrogram, the analysis revealed that chemoheterotrophy was the most prevalent activity, followed by anaerobic chemoheterotrophy, fermentation, human-associated functions, and aerobic chemoheterotrophy. We then performed an ANOVA analysis to examine the distribution of the four top categories of potential metabolic functions among the initial milk temperatures (see Supplementary Material Figure S5). Except for chemoheterotrophy, where samples with a temperature of 9°C before pasteurization showed only slight differences from the other two temperatures (Fig. S5A), cheeses from milk exposed to 7°C generally differed the most and showed the highest relative abundance (Fig. S5B, C, and D).

## Discussion

As a necessary premise, when evaluating sequencing-based results, it is important to recall that, as in all metagenomics and metabarcoding studies, the analyzed material is DNA. This does not necessarily mean that the obtained reads should entirely belong to cells that were viable or metabolically active at the time of sampling.

As anticipated, one of the purposes of this trial was to compare it with a previous temperature-shifting experimentation (Giagnoni et al. 2023), in which the cheesemaking operations were hosted in the same industrial facility, and the metabarcoding and bioinformatics protocols adopted were the same. The difference in the experimental design was that an average milk hygiene quality farm was used as a milking and milk-hosting facility, whereas a high milk hygiene quality stable handled the milk tank and managed its shifts in the cited report. This comparison is very relevant for the practical application of the cheese factory, because the chosen situation represents the average type of milk-contributing source encountered in the territory. Therefore, while the work of Giagnoni et al. (2023) can be considered pivotal to establish the baseline for these phenomena and to study responses in a more microbiologically controlled environment, the present trial more adequately represents the daily routine for that and many other cheese-making plants worldwide. The goal here was to analyze how possible deviations from a given refrigeration temperature were associated with differences in the DNA-inferable microbiota of the final products. Once again, the entire dairy processing chain was a real industrial setting, thanks to the industrial partner’s facilities, which managed the supply chain from primary steps to cheese production in their state-of-the-art plants.

An important detail of the technical setup of this experiment was the gradual and paced programming of the temperature rise. Another key point was the decision to perform the experiment in the same reservoir tank. After reaching each thermal point, the corresponding temperature was maintained for 12 days. Although milk was routinely refreshed daily, as customary in the dairy production chain, only the milk from the twelfth day after the temperature was set was used for the cheesemaking trials.

The three temperature conditions were therefore applied sequentially within a single storage tank rather than in independently replicated tanks. This choice necessarily introduced a trade-off in the experimental design, in which priority was given to the minimization of the technical variability, as opposed to the independent biological replication, which would have involved inter-factory trials.

The same-tank strategy was critical in allowing the tank habitat to develop a stabilized acclimatization that reflected the composition of the specific microbial population after a sufficiently extended conditioning phase. This practice avoided possible transient sharp successional surges in community composition and allowed for sampling once the new equilibria had stabilized. Otherwise, if the temperature had been shifted for just one day, stochastic day-by-day drift related to the milk components could have overwhelmed the deterministic temperature-driven effects we sought to investigate. The regular cleaning routine of the tank after milk removal was applied throughout. The temperatures increased between each experiment, starting from 4°C, then 7°C, and finally 9°C. The upper limit of 9°C was imposed by the substrate itself, because higher temperatures were found to cause consequences in fluidity. In this respect, in fact, as an additional finding, we observed that milk from average stables has a microbial load and composition conducive to coagulation risk during pasteurization when stored at higher temperatures. Interestingly, such behavior was not observed in previous literature reports with milk from top-quality stables, which can withstand thermal abuse up to 13°C (Giagnoni et al. 2023). Using, as mentioned, also in this trial, a serial rather than parallel approach (one reservoir instead of multiple ones) the same milk-collecting tank hosted incrementally changing temperatures over time. Due to this choice, the possible effects of randomly stochastic events linked to independent founder taxa were intended to be minimized. The fact that the three derived cheese communities maintained differences linked to each of the three serially realized temperatures, notwithstanding the common physical niche, suggests that a genuine differentiation, apparently tied to the sole temperature variable, had been obtained. An environmental contribution from the inevitable daily milk variability remains, nevertheless, always possible

Another reason for the preference for the same tank was related to the industrial context of interest. Indeed, events of accidental thermal abuse occur in production lines set to operate at a lower standard temperature; thus, when changes occur, they actually happen in the same reservoir. In general ecology, perturbations are events that affect an environment and alter its steady state, which is determined by pristine conditions in the same location. Therefore, the chosen setup was deemed useful from both an applied standpoint, which is of interest to the factory, and to gain inferences that benefit overall ecological knowledge.

The experimental design allowed us to reveal an effect that we have here referred to as an environmental legacy, in that it appears linked to the preceding communities. It needs to be understood that, as anticipated, this applies to a joint set of factors that include both the tank temperature and the milk load of the day, whose intrinsic daily variation could have added its contribution.

Beyond this clarification, by observing the composition of the downstream bacterial cheese communities, one could comment that the outcome was as if they had received the equivalent of a form of ‘*imprinting*’. The use of this term as an analogy and the use of quotes are, however, important to state that no evidence of causation can be claimed in this respect.

The first main difference between this trial and our prior one, in line with the intended premises, was noticed in the milk microbiological pattern regarding in-factory routine checks via agar plate culturing of colonies. The differences are summarized in Table 2.

**Table 2:** Milk microbiological parameters comparison.

	Giagnoni et al. 2023			This work		
Milk temperature	4°C	7°C	13°C	4°C	7°C	9°C
Total bacteria (CFU/ml)	10 500	12 000	12 500	95 000	150 000	1 500 000
Coliforms (CFU/ml)	200	500	1000	8000	25 000	70 000

Data are compared between the present study and a cited other report that had been run in a high-quality stable facility. Microbial abundances were assessed by culturable bacterial counts on specific media plates. CFU: Colony Forming Units.

Regarding the differences observed in the main microbial actors of the process, *Pseudomonas* is a major concern due to its involvement in off-flavor release through the production of volatile compounds and amino acid metabolites. Additionally, it produces thermotolerant proteolytic and lipolytic enzymes, including lipases, proteases, and lecithinases. These enzymes significantly reduce the quality and shelf life of protein-rich foods, such as milk and fresh dairy products (De Jonghe et al. 2010, Baruzzi et al. 2012, Raposo et al. 2016; Arslan et al. 2011,, (Craven and Macauly 1992), Ribeiro Junior et al 2018). As seen in Table 1, its presence in the milk was already conspicuous and clearly related to temperature in inverse proportions: more than 92% of the taxa at 4°C, 37% at 7°C, and finally, only 6.4% at 9°C. This led to high shares in the ensuing cheese (between 41% and 16%, respectively). In the higher-quality farm of the cited article (Giagnoni et al. 2023), the total bacterial load was 10–100 times lower (Table 2), and the maximum relative abundance of *Pseudomonas* in milk was 2.35% at 4°C. Its rise was only observed as a result of lowering the tank temperature from 13°C to 7°C, which is not featured in the present work, in which we experimented continuous increases and no reversions. That behavior is in line with *Pseudomonas*’ advantage in descending temperature scenarios, being a psychrotroph. In cheese, *Pseudomonas* rarely had an abundance over 1% in those prior trials of ours, reaching about 5% only with the aforementioned inverted gradient direction and lowered tank temperature. As an undesirable taxon, this comparison offers an interesting and technically counterintuitive consideration. While quality standards for milk storage are important, a colder temperature in an average facility would not produce the same results as in a top-quality farm setting. As our data show, in a less hygienic tank with a more competitive biota, a higher temperature could reduce the proportional outbreak that *Pseudomonas* would otherwise display. It needs to be also noted that, while the relative abundance of the various *Pseudomonas* instances was above 80% in milk at 4°C, and just around 4% in milk at 9°C (Table 1), in absolute terms, their numbers were still high as the latter milk had a 15-fold higher total bacterial load than the former one (Table 2). However, this does not equate to an expectedly comparable potential in proteolytic or lipolytic activities, because in the second condition (9°C) the *Pseudomonas* group lay outnumbered by the dominance of other taxa ready to compete for the same resources but to unfold different metabolic activities.

In the work from Giagnoni et al. (2023), which used milk from high-quality stables, psychrotrophic *Pseudomonas* was not a significant concern regardless of the milk storage temperature. However, in the present study, we found that it could become dominant. Nevertheless, this risk was greatly reduced by storing milk in a tank at a higher temperature. This links the results to a traditional piece of knowledge held by veteran dairy factory workers: the idea that, despite less hygienic stables and more coliforms in the milk in the past, the cheese outcome was more consistent and less uncertain. Conversely, cleaner, modern sites avoided coliforms, but this led to a higher occurrence of psychrotrophic *Pseudomonas* and less controlled proteolysis (M. De Noni, personal communication).

Further details on minor occurrences are discussed in the Supplementary Material section.

Concerning the possibility of disentangling inoculum legacy effects from habitat-driven selection processes occurring during ripening, we can point out that the rationale to consider the environment-derived microbial contribution as non-prejudicial to observe the effects of the milk lots was that the environment was common and its contribution fixed. The site of maturation was the same room. The ensuing habitat-driven selection processes are to be considered as exerted equally on all samples from the single common habitat. Upstream of that, the experimental design ensured that even the milk storage tank was the same for the three tested thermal levels, in order for the temperature to remain the only variable.

One of the most relevant pieces of evidence stemming from this analysis is that the effects of milk temperature are not necessarily reflected in a resemblance of community taxonomy between milk and cheese. This occurred at 4°C and 7°C, but not at 9°C. Rather, it appears to be a function of the independent coherency of successions that can occur to various extents in samples with the same initial milk temperature.

This observation has a central point of ecological relevance. Changes shown downstream by the biotic communities are not a function of taxonomy, but rather of the habitat that shaped them. In other words, habitat conditions (in this case, milk temperature) influence the subsequent endpoint taxonomy. However, the peaking order of taxa in terms of dominance proportions or presence could be maintained (4°C and 7°C) or completely altered (9°C) when comparing the initial state with the final one. After the initial habitat effect, the members of the final assemblages in each of the three groups of cheese samples descending from the three temperatures appeared to be simply drawn from those enduring the bottleneck of pasteurization and starter invasion. Then, they were passively driven by secondary succession, which led to a new habitat. This time, the habitat was common (milk had become cheese).

Some technical considerations for the correct interpretation of the metabarcoding data to infer ecologically relevant conclusions are provided at the end of the Supplementary Material in the section:

### Methodological framework and its suitability to extract ecological information

As previously mentioned, each of the three types of self-coherent, time-stable communities observed in cheese showed that they could result either compositionally similar to their corresponding “seeding” assemblage in milk (as in the cases of 4°C and 7°C) or profoundly dissimilar in taxonomic terms (as in the case of the 9°C milk), but yet clustering just as strongly as the other two. In this context, a difference is also found in species richness. Table 1 shows that the number of ASVs in milk at 7°C and 9°C was similar and higher than that yielded at 4°C. However, Fig. 2 shows that the Venn diagrams of cheese communities at 9°C had drastically lower diversity and fewer unique taxa.

Alpha-diversity analysis, supported by the Chao1 index (Fig. 3a) and the Venn diagram results (Fig. 2), revealed a noteworthy trend: samples stored at 7°C exhibited the greatest taxa richness. This finding was further substantiated by the analysis of differentially represented genera conducted in the context of three pairwise temperature comparisons (Fig. 6a). The convergence of these results reinforces the idea that the intermediate temperature of 7°C fostered the development of a microbial community with the highest taxa diversity across the tested intervals. Integrating these different approaches highlights the importance of temperature as a critical factor in shaping microbial diversity in this biotechnologically managed environment.

The key result, revealed by the present data, is depicted by the paired NMDS plots in Fig. 5 and backed up by the PERMANOVA analysis. Essentially, within these conditions, the original temperature to which the milk was exposed prior to cheesemaking showed the strongest association with variation in bacterial assemblage composition across the trial, despite subsequent 75°C pasteurization and the addition of a *Streptococcus thermophilus* starter culture. This phenomenon was consistently observed when the main species in milk remained dominant in cheese communities (milk stored at 4°C or 7°C) and when taxa leading milk compositional scores were fully substituted in cheese (milk stored at 9°C). The clustering of all replicates into coherent clouds in compliance with the starting milk temperature (Fig. 5a) demonstrates that the new cheese communities did not arise from random population drift bottlenecks, but rather from a constant legacy, particularly in the latter case. Inspecting the three groups of sample points in the ordination plot reveals that those from the 7°C value are the most scattered, consistent with the higher variability observed (richest in total and unique ASVs, as seen in Fig. 2’s Venn diagram) and the lower community dominance in those assemblages compared to those from 4°C and 7°C.

Examining the taxa that differentiate the three groups of communities descending from the three incremental milk temperature values (Fig. 6), besides those whose dynamics have already been discussed due to their numerical dominance (*Pseudomonas, Streptococcus, Achromobacter, Alkanindiges*, and *Lactococcus*), include *Halomonas*. This genus is associated with saline habitats but has been reported in cheese (Oguntoyinbo et al. 2018). This is explained by the 20% salt brine treatment and increased abundance above 0.1% in 7°C milk cheese samples but low abundance in samples from other temperatures. Another taxon that was not abundant but took advantage of the intermediate temperature was *Halanaerobium*. This genus is known to be salt- and alkali-tolerant (Brown et al. 2011). The residual presence of *Psychrobacter*, a close relative of the dominant genus *Acinetobacter*, is explained by its frequent association with raw milk (Olajide and LaPointe 2022). *Enhydrobacter* is a frequent species in raw milk (Guo et al. 2021), though it barely survives in dairy products (Oki et al. 2014). Members of the *Burkholderia-Caballeronia-Paraburkholderia* complex in milk are reported to be associated with mastitis (Wang et al. 2022). However, their abundance in milk or cheese was below 0.01% of the total, ranging around 20–40 reads per sample. These samples show the highest abundance in cheese from milk stored at 7°C. The genus *Staphylococcus*, which includes potential mastitis-associated species such as *S. aureus*, is another point of concern (Fagundes et al. 2010). However, as with the previous taxon, its occurrence in cheese microbiomes was below 0.01% of the reads. This taxon is usually inactivated by pasteurization, however. Furthermore, the ability to detect these taxa, even in trace amounts, supports the validity and sensitivity of the metabarcoding approach as a tool for inspecting dairy chain microbiota.

Few studies had so far addressed the effect of milk refrigeration temperature on the microbial community composition modulation, and those are essentially limited to specific cheese products that call for raw milk, as the Provolone Valpadana PDO (Zago et al. 2022), in which authors tested 10°C and 12°C for 15 h, and commented that the shifts in the dominant taxa, which also involved the increased shares of Lactobacillaceae. were considered not detrimental to the hygienic milk quality and advised the possible adoption of higher raw milk storage temperatures for such long-ripening cheese types, in which pasteurization is not part of the protocol.

## Conclusions

Upon testing the effect of two variables, the milk temperature in the collection tank and the duration of semi-fresh cheese ripening, the former was found to have a greater effect than the latter. The initial milk temperature ultimately appeared to have played a role in shaping the community structures across the process. The comparison of this work with the results of different trials (Giagnoni et al. 2023) also taught how the control of psychrotrophic cold-tolerant undesired biota, such as pseudomonads in milk collected from average stables not endowed with stringent top-quality hygienic standards, could even benefit from a deliberately raised milk tank storage temperature. These results suggest that the 4°C benchmark should not be assumed to be universally optimal without considering the production context and milk quality conditions.

Overall, this knowledge advantage empowers dairy industry professionals to optimize their production strategies, ensuring both product quality and process efficiency. Moreover, it underscores the importance of considering nuanced factors in decision-making processes within the cheesemaking business, promoting a more efficient and resource-effective approach to maintain a sustainable and profitable enterprise in this key sector of the global food manufacturing world.

## Supplementary Material

fnag046_Supplemental_File

## Data Availability

Sequence data have been deposited in Genbank under accession number PRJNA1395772.
