# Supplementary material for: The legacy of raw milk storage temperature is associated with cheese microbiome composition, notwithstanding pasteurization and starter addition
Source: FEMS Microbiol Lett. 2026 Apr 16;373:fnag046. doi: 10.1093/femsle/fnag046 (PMC13131215; doi:10.1093/femsle/fnag046)
Supplement: fnag046_Supplemental_File [file fnag046_supplemental_file.docx]

**Supplementary Material**

**The legacy of raw milk storage temperature is associated with cheese microbiome composition, notwithstanding pasteurization and starter addition**


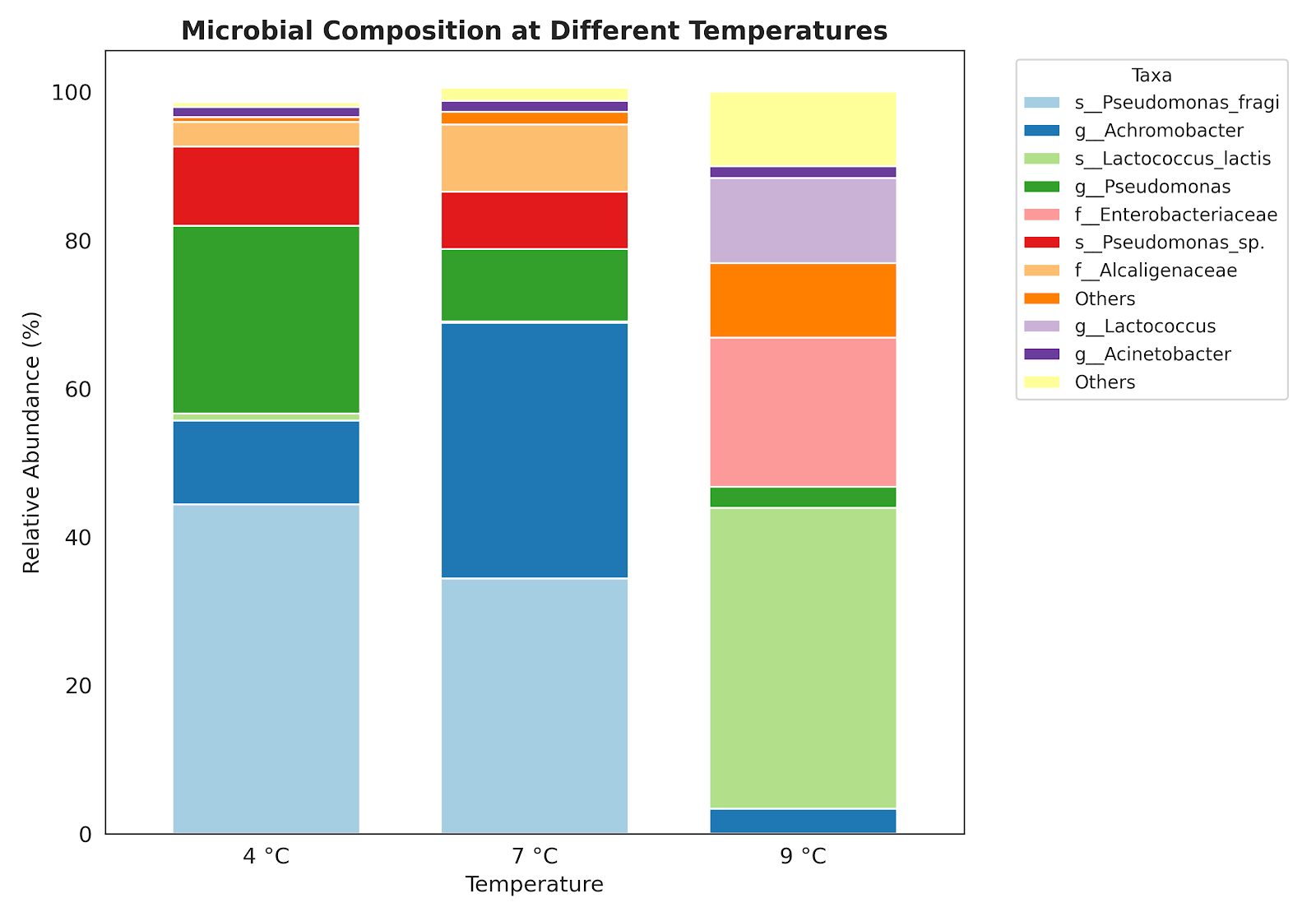


**Fig. S2. Relative abundance of the ten most abundant bacterial genera in milk as a function of its**

**storage temperature (4 °C, 7 °C, 9 °C).**

**
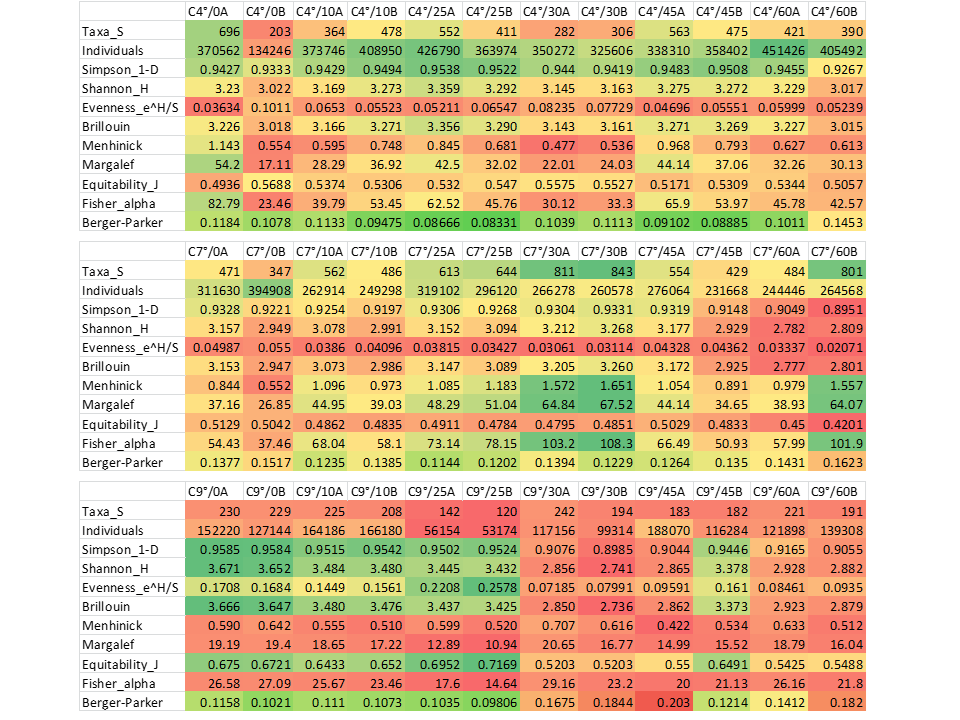
**

**Fig. S2. Detailed comparison of the sequencing quantitative results and corresponding ecological indexes.** Data refer to the communities detected in the cheeses at the different maturation stages. The samples came from the three raw milk storage temperatures prior to pasteurization, namely 4°C (top panel), 7° C (middle panel), and 9° C (bottom panel). Taxa _S indicates the number of different ASV (Amplified Sequence Variants)-defined taxa, and their sequence reads abundance for each sample (individuals) is reported. In the following rows, a series of classic community diversity measures are shown, including Shannon-Wiener H value, Simpson’s 1-D, Community Evenness (e^H/S), Brillouin Diversity Index, Menhinick richness index, Margalef Richness Index, Equitability index J, Fisher alpha Diversity Index, and Berger-Parker Dominance. The columns list the cheese samples of origin, using the following codes: Prefixes: C: Cheese. Infixes: Temperature (7 °C ad 9°C) at six maturity stages (1, 2, 3, 4, 5, 6) for the cheese samples. Suffixes: replicates A or B. Shades from green (higher value) to red (lower value) of the Microsoft Office Excel conditional formatting tool evidence abundance changes for every line (except for the Berger Parker dominace which has the opposite ecological meaning) from green (lower value) to red (higher value).

This figure displays the sequencing output in quantitative terms along with nine ecological indices that cover species diversity and community evenness. Besides the species diversity parameters already mentioned, other indicators were calculated. Community evenness (e^H/S) (Buzás and Hayek, 2005), Brillouin diversity index (Rothstein, 1956), Menhinick richness index (Menhinick, 1964), Margalef richness index (Margalef, 1958), equitability index J (Shannon, 1948), Fisher alpha diversity index (Fisher et al., 1943), and Berger-Parker dominance (Berger and Parker, 1979). In addition to the trends observed in Figures 2 and 3 regarding the inverse relationship between the number of taxa and alpha diversity indexes, a notable phenomenon emerges in the cheese produced from milk stored at 9 °C. There was a significant decline in most ecological indices, beginning between 25 and 30 days of maturation, irrespective of the number of sequence variants, which remained consistently low, yet stable.


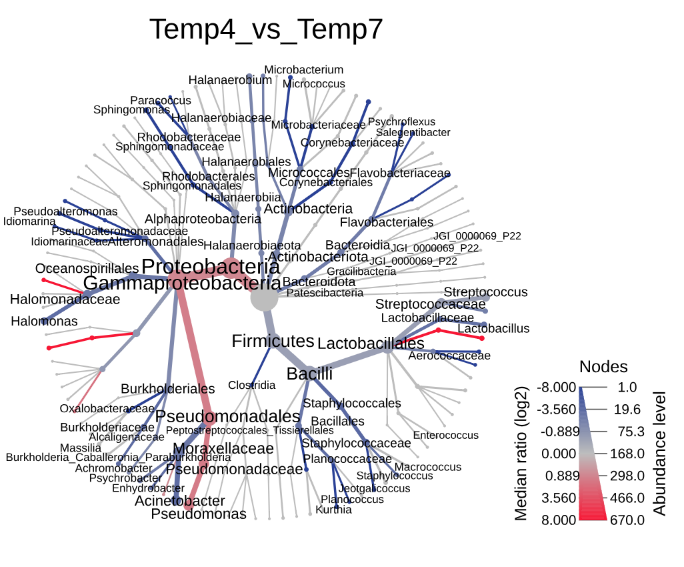


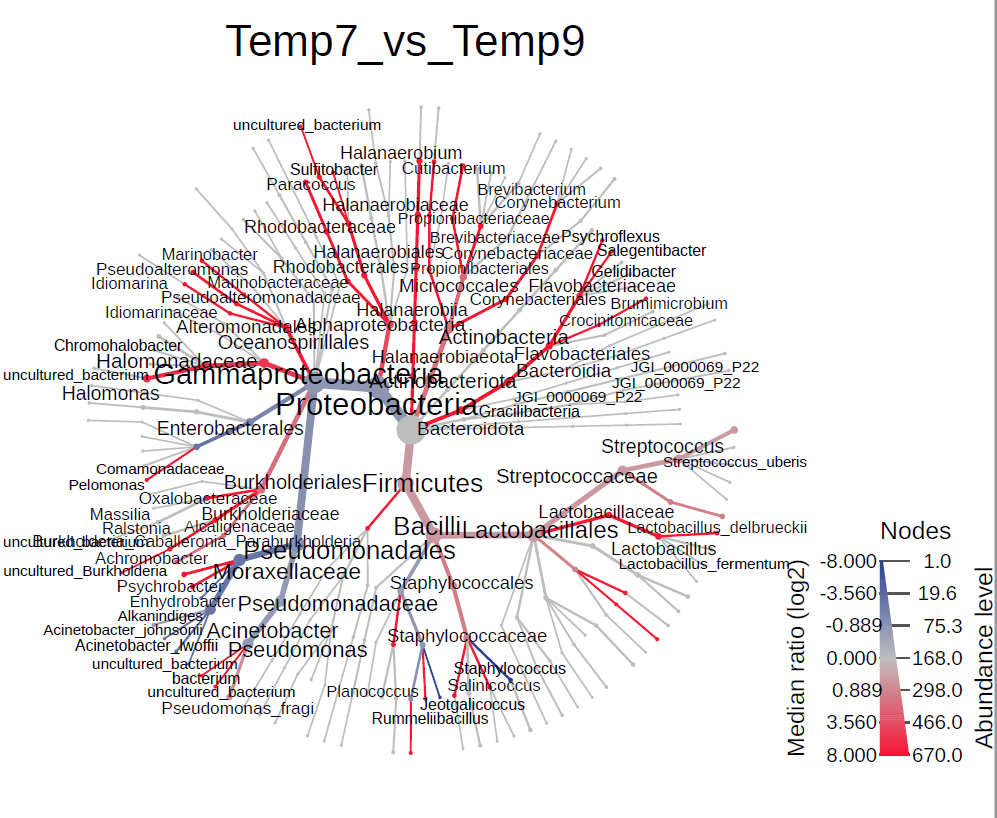

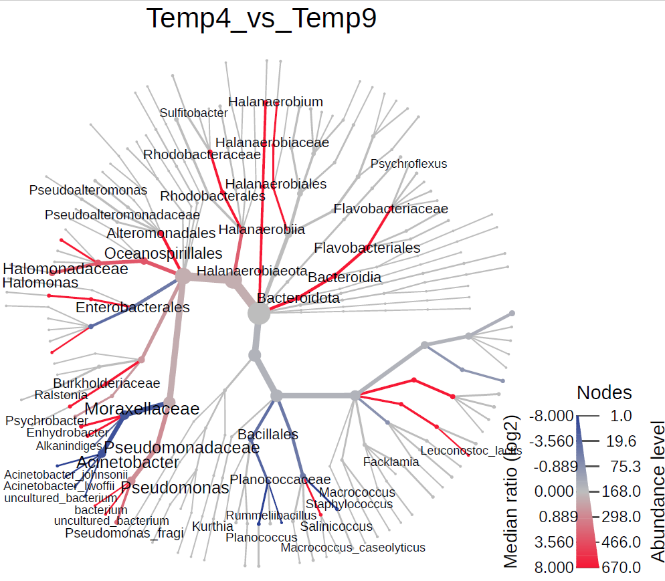


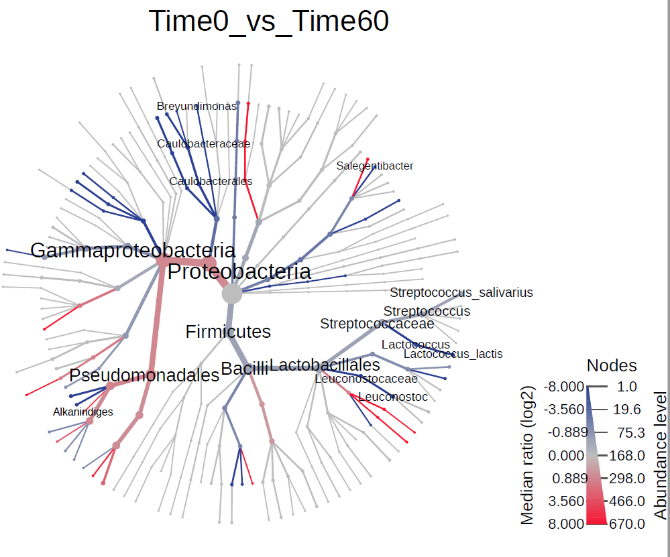


**Fig. S3 Heat tree analyses with comparison between cheese as a function of raw milk storage temperatures or cheese maturation time.** (Top left): 4°C vs 7°C; (Top right): 4°C vs 9°C; (Bottom left): 7°C vs 9°C; (Bottom right): Time zero vs. 60 days. Altered taxa are displayed by name at the corresponding node. Nodes follow the hierarchical taxonomy structure. A red branch indicates an increase in the first member of the comparison, while a blue branch indicates a decrease.


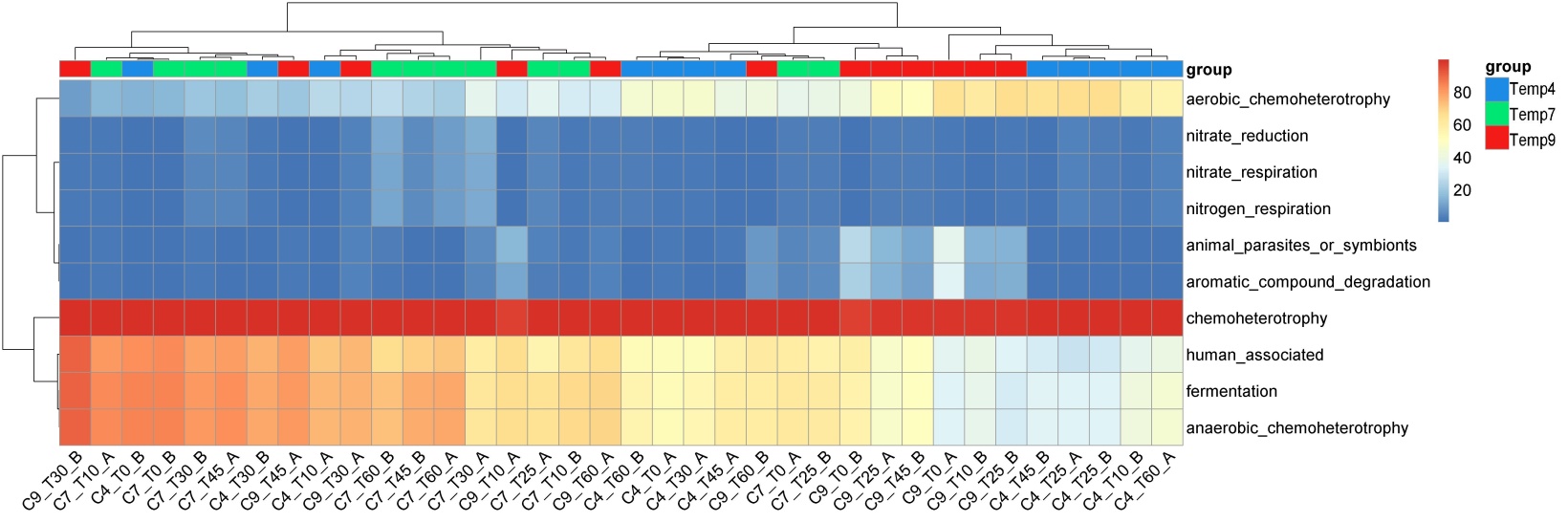


**Fig. S4. Heatmap** **from the FAPROTAX functional prediction analysis inferring the main putative metabolic processes of the microbial communities.** Prefix: C: Cheese. Infixes: Temperature 7 °C, 9°C, and 4°C followed by the six maturity stage time 0 days. 10 days, 25 days, 30 days, 45 days, 60 days. Suffixes: replicates A or B.


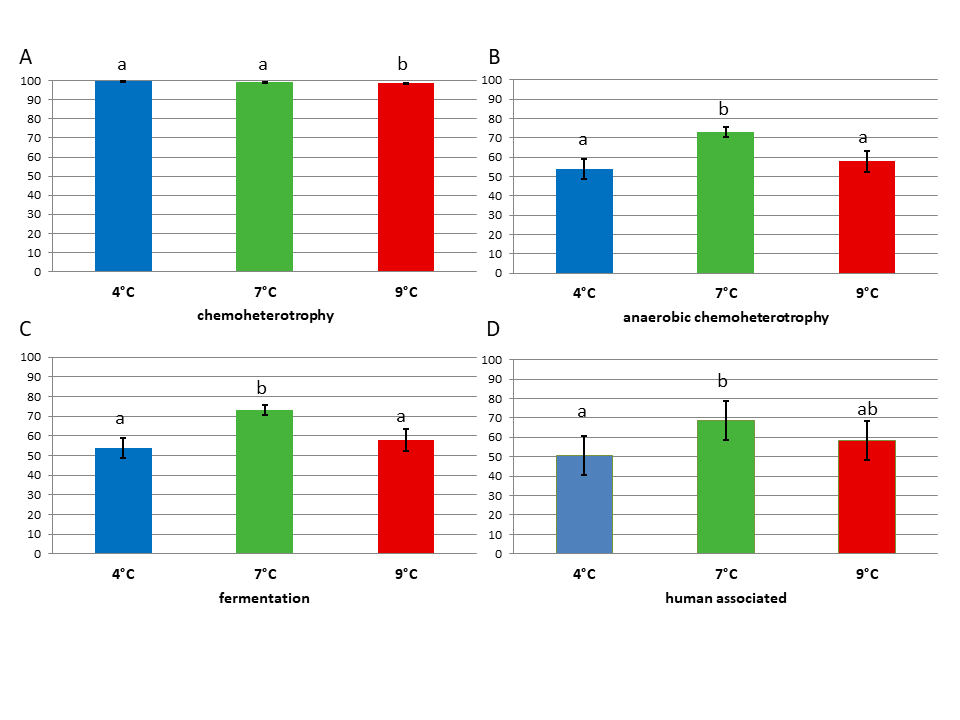


**Fig S5.** **Histogram bars of the most abundant potential metabolic functions predicted by the FAPROTAX analysis:** A): chemoheterotrophy, B): anaerobic chemoheterotrophy, C) fermentation, D) human-associated functions.

**Additional details in the taxonomical differences observed**

Another taxon considered a psychrotrophic dairy contaminant is *Acinetobacter* (Baruzzi et al., 2012; Ribeiro Jr. et al., 2018). In this case, unlike *Pseudomonas*, its presence in milk from the previous study was ten times higher than in the current one. In both studies, its prevalence in cheese increased with higher milk storage temperatures, though its concentration in milk remained stable throughout the tested temperature increases. In this respect, we must consider reexamining the psychrotroph definition for *Acinetobacter*, as no tests have verified its performance under milk storage temperatures different from the standard.

The third most abundant taxon observed in the present study was *Achromobacter*, which was below the detection limit in both milk and cheese in the previous trial. In milk, *Achromobacter* exhibited a "bell-shaped" occurrence, which was mirrored in cheese to a lesser extent. This behavior consisted of a peak at the intermediate temperature of 7 °C, flanked by similar values at each of the two extremes (Table 1, Figure 1). *Achromobacter* is a member of the Alcaligenaceae family, which also features additional top-scoring taxa in milk at 7 °C (Table 1). This suggests that *Achromobacter* would qualify as a specialist of the 7 °C temperature niche. However, it was outcompeted at a 3 degree-lower temperature (by *Pseudomonas* in both milk and cheese) and at a 2 degree-higher one (by *Acinetobacter* in milk and by *Lactococcus* and Enterobacteriaceae in cheese).

Regarding taxa that were not carried over in considerable proportions in the corresponding cheese lots despite their dominance in milk, very instructive examples arise from milk stored at 9 °C, which had the highest general bacterial load (over 1.5 million culturable units per mL, Table 2). It was taxonomically dominated by *Lactococcus* (almost 40%) and several members of Enterobacteriaceae and Enterobacteriales, including the genera *Enterobacter*, *Citrobacter*, *Serratia*, *Klebsiella*, *Hafnia*, and *Rahnella*, which collectively accounted to over 40%. Despite this, neither *Lactococcus* nor any Enterobacteriaceae ended up with abundances higher than 0.03% in the resulting cheese.

Such scarcity was observed from the beginning of the cheese maturation process and remained consistent over time. Some explanatory factors that can help interpret why some taxa would be present in both milk and cheese, as described above, while others would technically become extinct include: 1) the variable efficiency of the pasteurization treatment, which can affect different species unevenly; 2) the varying durability of DNA proficient in sequencing inside heat-inactivated cells, as well as that oozing out of lysed ones; and 3) the numerical competition exerted by cells and spores that reproduce in cheese and have endured pasteurization, as well as those of the added *Streptococcus* starter. In the case of *Lactococcus* and the starter, which are both members of the same family, the higher fitness of the selected inoculant for the cheese habitat could play an even stronger role. Commenting further on the behavior of the starter strain, which was added in equal amounts to all cases, it displayed its worst performance in terms of abundance (mean of 55.69%) at the lowest milk refrigeration temperature of 4 °C (which is nevertheless the temperature traditionally adopted by the dairy industry), where competition with the spoilage-proficient *Pseudomonas* was observed. The highest starter abundance in cheese (75.13%) occurred when the milk came from the 7 °C tank. In those from the 9 °C tank, the value attained was 61.40%. In light of the changes in community structure discussed here, particularly the drastic disappearance of *Lactococcus* and Enterobacteriaceae, it is important to note that despite these major taxonomic shifts, the Bray-Curtis similarity levels of each community, on which the NMDS is based, still form tight clusters that allow to trace each cheese community back to the temperature at which the originating milk was exposed, as Fig. 5 shows.

**Methodological framework and its suitability to extract ecological information**

We have shown that the effects of milk temperature are profound, but remarkably, they do not necessarily consist of a conserved taxonomy between milk and cheese. It occurred at 4 °C and 7 °C, but not at 9 °C. The habitat condition (milk temperature) determined the final taxonomy independently of the initial one that could be maintained or restructured. This observed difference is also useful evidence that dispels a possible criticism faced by metagenomics in general. The methodology is based on a culture-independent strategy and a life-independent event because the in vitro PCR enzymatic reaction works on naked DNA extracted from cells inevitably killed during lysis required by the protocol. In these microbial metabarcoding studies, it is impossible to distinguish between sequencing reads that come from relic DNA belonging to cells that were already dead in the environmental sample and those that come from cells that were alive but were killed by the sequencing methodology protocol itself. The 75 °C pasteurization kills the sensitive fraction of the population but does not destroy their DNA, which could still be compatible with PCR amplification. The result of the chain reaction itself demonstrates that repeated cycles of DNA denaturation at 95 °C are the basis of the PCR functioning principle. However, the data themselves offer an answer to this possible argument in the present experiments by looking at the observed differences between the milk and cheese communities. If the result were biased by the bulk of DNA, regardless of the live or dead status of the proprietary cells in the environment before sampling, the dominant members of the cheese community, besides the added starter, would mirror the proportions observed in their originating milk. Conversely, if that were the case, the cheese produced from milk with a *Lactococcus* and Enterobacteriaceae sum of over 80% of the sequences, should reflect that pattern. However, none of the samples in this line have values over 0.03% for either taxon. Therefore, the issue of possible bias in next-generation sequencing due to extracellular DNA fragments that are no longer associated with the vital activities of the originating bacterium is not applicable here. It is possible to distinguish that the aforementioned concern could be valid when sampling environments such as soils and sediments, where most microbes are quiescent and dead cells and remnant DNA can be protected from consumption and degradation by absorption onto clay particles or due to scarcity of water, minerals, and other nutrients, as well as other factors that apply to such life-limiting contexts. Conversely, in fully organic matrices such as milk and cheese, which are growth-supportive and successionally open habitats, as well as in other physiology-driven environments such as animal guts and bioreactors, it is highly likely and well-documented that cells of succumbing species, along with their liberated DNA, are promptly consumed by thriving, multiplying species, thus avoiding the risk of blurred and obscured results from the prior stage. Furthermore, it must be noted that the microbial biomass in milk is much lower than in cheese. Thus, a high proportion of a particular bacterium in milk does not guarantee a high proportion of it in the resulting cheese.

**Cited literature**

Baruzzi F, Lagonigro R, Quintieri L, Morea M, Caputo L. Occurrence of non-lactic acid bacteria populations involved in protein hydrolysis of cold stored high moisture Mozzarella cheese. Food Microbiol 2012; **30**:37–44. <https://doi.org/10.1016/j.fm.2011.10.009>

Berger WH, Parker FL. Diversity of planktonic foraminifera in deep-sea sediments. Science 1970; **168**:1345–1347. <https://www.science.org/doi/10.1126/science.168.3937.1345>

Buzas MA, Hayek LAC. On richness and evenness within and between communities. Paleobiology 2005; **31**:199–220. [https://doi.org/10.1666/0094-8373(2005)031[0199:ORAEWA]2.0.CO;2](https://doi.org/10.1666/0094-8373(2005)031%5b0199:ORAEWA%5d2.0.CO;2).

Fisher RA, Corbet AS, Williams CB. The relation between the number of species and the number of individuals in a random sample of an animal population. J Anim Ecol 1943; **12**:42–58. <https://doi.org/10.2307/1411>

Margalef R. Information theory in ecology. General Systems 1958; **3**:36–71. http://hdl.handle.net/10261/284346

Menhinick EF. A comparison of some species-individuals diversity indices applied to samples of Ribeiro Júnior JC, De Oliveira AM, Silva FDG, Tamanini R, De Oliveira ALM, Beloti V. The main spoilage-related psychrotrophic bacteria in refrigerated raw milk. J Dairy Sci 2018; **101**:75–83. <https://doi.org/10.3168/jds.2017-13069>

Rothstein J. Science and Information Theory. Academic Press, New York, 1956, 320 pp. Science 1956; **124**:492–493. <https://doi.org/10.1126/science.124.3220.492.b>
